# Dementia as a predictor of care-related quality of life in informal caregivers: a cross-sectional study to investigate differences in health-related outcomes between dementia and non-dementia caregivers

**DOI:** 10.1186/s12877-018-0885-1

**Published:** 2018-08-23

**Authors:** Nina Karg, Elmar Graessel, Ottilie Randzio, Anna Pendergrass

**Affiliations:** 1 Department of Psychiatry and Psychotherapy, Center for Health Service Research in Medicine, Friedrich-Alexander-University Erlangen-Nuremberg, Schwabachanlage 6, D-91054 Erlangen, Germany; 2 Medical Service of Compulsory Health Insurance Funds (MDK) of Bavaria, Haidenauplatz 1, D-81667 Munich, Germany

**Keywords:** Home care, Informal caregivers, Dementia, Caregiver health, Care-related quality of life

## Abstract

**Background:**

The objectives of this study with a large sample of informal caregivers (CG) were a) to compare health-related outcomes of CGs caring for a patient with dementia and those caring for a relative with another chronic disease and b) to check whether dementia is a predictor of CG’s care-related quality of life (QoL) in CarerQoL-7D.

**Methods:**

This cross-sectional study involved self-reported data from 386 informal CGs who applied for an initial grade or upgrade of the care level of the care recipient at the Medical Service of Compulsory Health Insurance Funds of Bavaria (Germany). By obtaining data this way, systematic biases often associated with the acquisition of CGs were prevented. Bivariate and multiple analyses were conducted using a univariate covariance model (ANCOVA).

**Results:**

Bivariate analyses showed significantly higher levels of subjective burden and lower QoL in the dementia group. No significant differences were found in terms of physical health and depressiveness, though there was a tendency suggesting higher levels of depressiveness in dementia CGs. Multiple analysis explaining QoL by dementia status after controlling for CG’s sex, age and employment status revealed a significant effect of dementia, suggesting caregiving for a dementia patient was associated with lower QoL.

**Conclusions:**

Results of the study suggest that caring for a relative with dementia is associated with poorer health, i.e. greater levels of subjective burden and depressiveness, and predicts lower QoL in CGs. These findings emphasize the importance of specific interventions aiming to support informal CGs of dementia patients.

## Background

In 2013, 71% of all care recipients (CR) legally classified as dependent on long-term care in the Federal Republic of Germany lived in private households and in 92% of the cases they received daily assistance from private individuals providing informal care on a non-professional base [1, 2]. By accepting the responsibility for a care-dependent family member, informal caregivers (CGs) are faced with a variety of new challenges that transform the individual life. Numerous studies have examined the effects of caregiving on CG’s health and demonstrated that CGs are at higher risk of jeopardizing both their physical and mental health compared to non-caregiving subjects of the same age and population (e.g. [3, 4]). Pinquart and Sörensen [4] found significantly higher levels of perceived strain in CGs compared to non-caregivers, which is associated with greater physical health problems and a higher likelihood of developing psychological and psychiatric morbidities, as well as higher mortality rates [5–7].

CGs of dementia patients are often called “the invisible second patients” [8], because dementia caregiving often exceeds the demands associated with caregiving for solely physically impaired CRs. Caregiving for a person with dementia is associated with several stressors that are specific to dementia and increase the likelihood of the development of chronic stress and associated adverse outcomes on CG health. The progress of the dementia disease is characterized by an unpredictable and uncontrollable deterioration of various cognitive, affective and eventually physical abilities. Deepening cognitive decline as well as the patient’s loss of physical capabilities lead to an overall decreased functional level associated with deficits in activities of daily living (ADL) and instrumental activities of daily living (IADL). The result is that dementia CGs must often spend more hours on caregiving compared to non-dementia CGs [9]. A number of studies have suggested that greater impairment in ADL and IADL, lower functional level and greater total care time are associated with higher levels of CG stress, subjective burden, depressiveness and lower physical health in CGs [10, 11]. Furthermore, caregiving literature has highlighted the importance of CRs’ cognitive impairment, which is more common amongst dementia patients than amongst non-dementia patients [12], as a cause of worse psychological well-being and mental health as well as physical morbidity in CGs [13, 14]. Cognitive decline and changes in personality commonly present as so-called disruptive behaviors, incomprehensible, disoriented or demanding patient behaviors. These have been found to be more common in dementia than in other forms of illness and have consistently been linked to greater distress, perceived burden and depressive symptoms in CGs [6, 10–14].

To date, a large number of studies have been conducted examining psychological and physical health of CGs of dementia patients and those caring for a person with functional impairment from another type of chronic illness. Dementia caregiving status was found to be a factor associated with higher levels of stress and subjective burden, and was linked to worse overall mental health outcomes like higher psychological and psychiatric morbidity [10, 15–17]. With respect to the impact of dementia caregiving on CG’s physical health results have not always been clear, but several studies revealed low levels of physical health and an increased risk of various health problems in CGs caring for dementia patients [8, 17–19]. Ory and colleagues [20] found significantly more physical strain in dementia CGs compared to those caring for elderly people with relatively unimpaired cognitive performance. However, the aforementioned studies relied to a great extent on rather small convenience samples that were recruited primarily from CG support groups or Alzheimer’s associations and therefore may be susceptible to systematic recruitment biases. More representative, unbiased samples with a greater number of participants would be essential to allow for more reliable and definite conclusions about the health-related consequences of caregiving. For this reason, the first aim of the current study was to investigate in a large, representative sample differences in health-related variables between those two subgroups of CGs.

Furthermore, a significant portion of previous research primarily focused on particular health-related aspects of informal caregiving, not taking into account the total impact of caregiving activity on the CG’s overall quality of life, from now on referred to as care-related QoL. Different approaches have been made to find an appropriate definition and measure for the concept of QoL in the context of informal caregiving, but due to the multidimensional nature of this concept, no consensus has yet been reached. Some research has focused on physical and mental well-being as measures of health-related QoL (e.g. [21, 22]), but according to the WHO [23] health means not only a state of complete physical and mental well-being, but social well-being as well. Thus, not only physical and mental health, but also social and economic factors as well as familial interactions must be taken into account when discussing an individual’s QoL [24, 25]. In consideration of this, Brouwer and colleagues [26] developed the CarerQoL, an instrument for the assessment of care-related QoL in informal CGs. The CarerQoL captures different aspects of the care situation affecting key domains in a person’s life and at the same time allows for an evaluation of the impact of caregiving activity on the CG’s overall QoL. Some attempts have been made to establish determinants of CG’s QoL, finding that lower QoL levels in informal CGs seem to be associated with e.g. greater severity of the patient’s behavioral problems, higher levels of CG burden and greater levels of depressiveness (e.g. [25–28]). In line with these findings, Srivastava and colleagues [29] found low levels of self-reported QoLin key CGs of dementia patients. However, this study did not involve a control group of informal CGs caring for patients with other chronic diseases. In their systematic review, Farina and colleagues [30] tried to identify factors predicting QoL in CGs of people with dementia and found that CG’s better physical health, mental and emotional well-being (i.e. lower levels of depression, anxiety and perceived burden), greater CG independence, self-efficacy and coping skills were related to CG’s QoL. With the exception of these findings, factors associated with QoL of CGs of dementia patients have not yet been examined to a satisfactory extent. Furthermore, no direct comparisons exist between dementia and non-dementia CGs. As previously shown, dementia CGs appear to be exposed to a greater variety of strains as well as to suffer from worse physical and mental overall health compared to non-dementia CGs. As more domains of the CG’s personal life are potentially affected by dementia caregiving, it can be assumed that CGs’ care-related QoL is more strongly affected in dementia CGs than in non-dementia ones. For this reason, the second aim of the current study was to examine whether caring for a person with dementia affects care-related QoL in informal CGs.

We hypothesized that

- CGs caring for CRs with dementia would report worse physical and mental health and lower levels of care-related QoL than CGs caring for CRs with other chronic diseases
- lower levels of the CG’s care-related QoL are attributable to the CR’s dementia illness

## Methods

### Research design and participants

This study is a cross-sectional study involving self-reported data. Between October 2014 and April 2015 1700 self-report questionnaires were handed out to informal CGs upon application for an initial grade or upgrade of the care level^1^ of the CR at different centers of the Medical Service of Compulsory Health Insurance Funds (MDK)^2^ all over Bavaria. 443 participants (26.1%) returned the questionnaires and thereby declared their consent with the utilization of their data for scientific evaluation. *Inclusion criteria* were a) providing care for someone with chronic care needs and b) CR 65 years or older. *Exclusion criteria* were a) lacking information about the presence or absence of CR’s dementia and b) providing care for own children. 57 participants (13%) did not meet the conditions and thus a final total of 386 participants were included in statistical analyses.

### Measures

#### Independent variables

Demographic and background characteristics recorded were the CG’s and CR’s age and gender, CG’s employment status and educational attainment, the relation between the relationship between the CG and CR, living situation, duration of care, total daily care time,^3^ medical cause of care dependency and comorbidities (including dementia) and support received by the CG.

Physical complaints were measured using the short form of the Giessen Symptom List (GBB) [31], a reliable and valid self-assessment measure for psychosomatic health complaints. The short form consists of 24 items, all of which are measured along a five-point Likert scale (0 = ‘not at all’ to 4 = ‘yes, absolutely’), that evaluate physical complaints on four subscales: exhaustion, gastrointestinal, musculoskeletal and cardiovascular complaints. By computing a sum score with a possible range between 0 and 96, the general impact of somatic complaints is assessed. For reasons of clarity, percentile ranks (*PR*) (median and median absolute deviation^4^) were calculated for descriptive analysis. Further analyses were carried out with the raw sum score.

To assess perceived burden, the Caregiver Strain Index (CSI) [32] was administered. The 13-item self-report questionnaire measures financial, physical, psychological, social and personal strain using a dichotomous answering format (0 = ‘no’, 1 = ‘yes’) with higher scores corresponding to higher subjective strain. Given a possible range from 0 to 13, a final score of 7 or higher reflects a considerable level of stress in the CG’s life.

The Patient Health Questionnaire-9 (PHQ-9) [33] is a reliable and valid screening measure that evaluates the intensity of self-reported depressive symptoms over the last two weeks on a 4-point-scale (0 = ‘not at all’ to 3 = ‘nearly every day’). Scores for the 9 items are summed (maximum of 27), with higher scores corresponding to greater severity of depressiveness and a total score > 9 indicating moderate or severe symptoms.

The 5-item subscale ‘positive aspects’ of the Berlin Inventory of Caregivers’ Burden - Dementia (BIZA) [34] was used to measure positive aspects of informal caregiving, so-called benefits, on a 5-point Likert scale (0 = ‘never’ to 4 = ‘always’). With a possible maximum sum score of 20, higher scores indicate that the CG perceived benefits to a greater extent.

#### Outcome variable

Care-related QoL in informal CGs was assessed using the *CarerQoL-7D* scale of the CarerQoL [26]**,** a reliable and valid [35]self-report instrument measuring care-related QoL on two distinct scales. The *CarerQoL-7D* covers seven personal key domains being affected by the caregiving activity (care-related fulfillment, a mental and a physical health dimension, a social dimension and perceived support, a relational dimension, and financial security) while the *CarerQoL-VAS* allows for an evaluation of the CG’s general level of happiness on a single-item visual analogue scale (VAS). Due to conceptual^5^ and methodological^6^ reasons, only the comprehensive set of the 7 care-related dimensions of the CarerQoL-7D was included in the survey questionnaire. Item responses are recorded on a 3-point scale (0 = ‘no’ to 2 = ‘a lot’), resulting in a sum score with a possible range between 0 and 14. Higher scores are associated with higher care-related QoL in informal CGs; thus the CarerQoL adequately reflects care-related QoL and provides a good overview of the impact of informal care on the CG [26].

### Statistical analysis

Descriptive statistics were computed to illustrate sample characteristics in the terms of frequencies, means (*M*) and standard deviations (*SD*) of the variables.

As underlying assumptions for parametric tests were met (independence, normality and homogeneity of variance), independent t-tests and chi-squared tests were conducted to check for group differences in variables of interest between CGs caring for a dementia patient (dementia group) and those caring for relatives whose care dependency had other causes (non-dementia group).

In the next step, bivariate analyses, including Pearson’s r correlations and independent t-tests, were performed to investigate the association between characteristics of the CG, CR and the care situation and CGs’ self-reported QoL. Variables significant at the bivariate level were included as covariates in the initial multiple model.

The multiple analysis was conducted using a univariate covariance model (ANCOVA) with care-related QoL as the dependent variable, ‘Dementia’ as the fixed factor and all variables which correlated significantly with care-related QoL as covariates.Collinearity statistics were examined in advance to ensure there were no issues of multicollinearity. In an iterative approach, variables not significant at the bivariate level were added one after another to the initial model, checking whether they contributed significantly to the explained variance of QoL in the multiple model over and above the variables already included. Variables with additional explanatory power were added as further covariates into the final model.

For all analyses, a Type 1 error probability (alpha) of less than 5% was considered to constitute statistical significance. Statistical analyses were performed with SPSS v.21.

## Results

Table 1 provides the results of the descriptive analysis, including the group comparison between the two subgroups dementia vs. non-dementia in all of the variables.

**Table 1:** Sample characteristics (*N = 386*)

	**Dementia** *(n = 153)*	**Non-Dementia** *(n = 233)*	***p***	**Total sample** *(N = 386)*
Caregiver (CG)				
Age (yrs.), *M (SD)*	60.8 *(13.0)*	61.6 (11.6)	*0.503* ^*a*^	61.3 *(12.2)*
Sex: Female, *N* (*%)*	122 *(80)*	173 *(74)*	*0.214* ^*b*^	295 *(76)*
Educational attainment (yrs.), *M (SD)*	10.8 *(2.7)*	10.7 *(2.6)*	*0.874* ^*b*^	10.8 *(2.6)*
Employment status: Employed, *N* (*%*) ^*c*^	57 *(37)*	100 *(43)*	*0.268* ^*b*^	157 *(41)*
Relation: Care recipient is… *N (%)*			*0.310* ^*b*^	
- mother/father (in-law)	94 *(61)*	140 *(60)*		234 *(61)*
- spouse	50 *(33)*	86 *(37)*		136 *(35)*
- other	9 *(6)*	7 *(3)*		16 *(4)*
Received support, *N* (*%)* ^*d*^	125 *(82)*	181 *(78)*	*0.341* ^*b*^	306 *(79)*
Subjective burden (*CSI*), *M (SD)*	7.9 *(2.6)*	7.1 *(2.9)*	*0.004* ^*a*^	7.4 *(2.8)*
QoL (*CarerQoL*), *M (SD)*	7.4 *(2.8)*	8.1 *(2.7)*	*0.013* ^*a*^	7.9 *(2.8)*
Depressiveness (*PHQ-9*), *M (SD)*	8.5 *(5.0)*	7.6 *(5.1)*	*0.094* ^*a*^	8.0 *(5.1)*
Benefits (*BIZA*), *M (SD)*	12.4 *(3.9)*	12.2 *(3.7)*	*0.726* ^*a*^	12.3 *(3.8)*
Physical complaints (*GBB*), PR, *MDN (MAD)*	80 *(21.0)*	74 *(23.5)*	*0.132* ^*a*^	76 *(22.7)*
Care recipient (CR)				
Age (yrs.), *M (SD)*	82.8 *(7.0)*	81.4 (7.5)	*0.063* ^*a*^	81.9 *(7.3)*
Sex: Female, *N* (*%)*	101 *(66)*	141 *(61)*	*0.275* ^*b*^	242 *(63)*
Number of morbidities (incl. dementia), *M (SD)*	2.88 *(1.68)*	2.43 *(1.50)*	*0.006* ^*a*^	2.60 *(1.58)*
Morbidities: Major disease groups (ICD-10), *N* (*%)* ^*e*^				
- II: Neoplasms	6 *(4)*	38 *(16)*		44 *(11)*
- IV: Endocrine, nutritional and metabolic diseases	33 *(21)* ^*f*^	53 *(23)*		86 *(22)*
- V: Mental and behavioral disorders	5 *(3)*	7 *(3)*		12 *(3)*
- VII: Diseases of the eye and adnexa	5 *(3)*	14 *(6)*		19 *(5)*
- IX: Diseases of the circulatory system	48 *(31)*	94 *(40)*		142 *(37)*
- X: Diseases of the respiratory system	7 *(5)*	11 *(5)*		18 *(5)*
- XI: Diseases of the digestive system	2 *(1)*	5 *(2)*		7 *(2)*
- XII: Diseases of the skin and subcutaneous tissue	1 *(1)*	1 *(0)*		2 *(1)*
- XIII: Diseases of the musculoskeletal system and connective tissue	14 *(9)*	41 *(18)*		55 *(14)*
- XIV: Diseases of the genitourinary system	3 *(2)*	12 *(5)*		15 *(4)*
Care situation				
Living situation: Together, *N* (*%)*	108 *(71)*	162 *(70)*	*0.824* ^*b*^	270 (70)
Duration of care (mo.), *M (SD)*	52.4 *(41.2)*	53.1 *(57.4)*	*0.892* ^*a*^	52.8 (51.5)
Total care time (hrs./day), *M (SD)* ^*g*^	11.9 *(4.2)*	10.8 *(4.1)*	*0.010* ^*a*^	11.2 *(4.2)*

*M* mean, *SD* standard deviation, *PR* Percentile Ranks, *MDN* Median, *MAD* Median absolute deviation, *CSI* Caregiver Strain Index (scores from 0 to 13), *CarerQoL* CarerQoL-7D (scores from 0 to 14), *PHQ-9* Patient Health Questionnaire-9 (scores from 0 to 27), *BIZA* Berlin Inventory of Caregivers’ Burden - Dementia (scores from 0 to 20), *GBB* Giessen Symptom List

^a^t-test

^b^chi-square test

^c^min. 7 yrs. (no compulsory school leaving certificate)- max. 18 yrs. (university degree)

^d^CG receives formal or/and informal support related to caregiving

^e^One or more diseases of the disease group have been reported; disease groups regarding unspecific impact factors on health (group 21) and abnormal clinical findings (group 18) are not included in the ranking

^f^not including dementia

^g^Consisting of time spent on activities of daily living (*ADL*), instrumental activities of daily living (*IADL*) and supervision of the CR (*SUVI*) by the CG

Informal CGs were on average 61 years old; three quarters were female; 40% were still employed and the majority had a moderate educational level. Most of the CGs were caring either for their parents (or in-laws) or spouse, who they lived together with in 70% of the cases. During an average duration of care of 4.5 years 80% of the CGs received informal or formal support. CRs mean age was 82 years, and more than half were female. CRs in the full sample were diagnosed with three illnesses (diagnoses according to the ICD-10 [36], including dementia) on average. Dementia patients reported significantly more morbidities (two additional comorbidities apart from dementia) than those of the non-dementia group (two morbidities). The prevalence of the ten major disease groups being most frequent in the age 65–80 group in Germany as a whole [37] are also shown in Table 1, with the numbers referring to our sample.

There was no evidence for further significant differences between the two subgroups with regard to the described characteristics, with the exception of total care time: CGs in the dementia group reported a significantly higher amount of time compared to the non-dementia group.

In terms of subjective burden, CGs in both groups experienced a considerably high level of strain (CSI score > 7), with the dementia group (*n* = 81 (53%) CSI > 7) reporting significantly higher burden scores than the non-dementia group (*n* = 110 (47%) CSI > 7). Both groups reported medium levels of care-related QoL on average (CarerQoL: 8 of 14 points, range: 1–14), again with dementia CGs indicating lower levels than CGs in the non-dementia group *(M = 7.4, range: 1–14* vs. *M = 8.1, range: 2–14).* Depressiveness scores in both groups indicated low to mild levels of depressiveness (PHQ < 9), with a tendency (though no significant difference) for the dementia group to have higher levels of depressiveness (*n* = 601 (39%) PHQ > 9 vs. non-dementia group: *n* = 73 (31%) PHQ > 9). Altogether, CGs reported psychosomatic health complaints (GBB median *PR:* 76) higher than in the average population (GBB median *PR:* 50) with no significant difference between the dementia and non-dementia group. In terms of benefits, both groups experienced positive aspects of caregiving to the same extent (BIZA: 12 of 20 points), indicating that the likelihood of experiencing benefits is higher than that of not experiencing any positive aspects of caregiving.

Bivariate correlational analyses revealed significant negative correlations between care-related QoL (CarerQoL)and depressiveness (PHQ-9), perceived burden (CSI) and physical complaints (GBB), suggesting that a greater subjective burden and physical complaints as well as lower levels of depressiveness were associated with higher levels of care-related QoL (see Table 2).

**Table 2:** Associations of Variables of Interest with QoL (*CarerQoL*) (*N = 386*)

	Test statistic	*p*
Depressiveness (*PHQ-9*)	*r = −.705* ^*a*^	*< 0.001*
Subjective burden *(CSI)*	*r = −.648* ^*a*^	*< 0.001*
Physical complaints *(GBB)*	*r = −.668* ^*a*^	*< 0.001*
Sex CG	*t = − 3.146* ^*b*^	*0.002*
Employment status	*t = 3.497* ^*b*^	*0.001*
Sex CR	*t = 1.552* ^*b*^	*0.122*
Living situation	*t = − 0.992* ^*b*^	*0.322*
Received support	*t = −1.121* ^*b*^	*0.263*
Relation ^c^	*t = −0.299* ^*b*^	*0.765*
Educational attainment	*r = −.083* ^*a*^	*0.102*
Duration of care	*r = −.017* ^*a*^	*0.738*
Total care time	*r = −.046* ^*a*^	*0.365*
Age CG	*r = .039* ^*a*^	*0.449*
Age CR	*r = .005* ^*a*^	*0.918*
Benefits (*BIZA*)	*r = −.030* ^*a*^	*0.554*

*CarerQoL* CarerQoL-7D, *CSI* Caregiver Strain Index, *PHQ-9* Patient Health Questionnaire-9, *GBB* Giessen Subjective Complaints List, *BIZA* Berlin Inventory of Caregivers’ Burden – Dementia

^a^Pearson product moment correlation, two-tailed

^b^t-test

^c^dichotomized (spouse vs. non-spouse)

As multicollinearity analyses revealed significantly high intercorrelations (*r* ≥ 0.5) between these variables, only depressiveness, which showed the strongest association with care-related QoL, was included in the final multiple model.

Furthermore, there was a significant difference in CG QoL depending on the CG’s sex and employment status, indicating being male (*M = 8.65, SD = 2.85* vs. female: *M = 7.62, SD = 2.70*) and unemployed (*M = 8.25, SD = 2.91* vs. employed *M = 7.29, SD = 2.44*) were associated with higher levels of QoL. Hence employment status and caregiver’s sex were included in the multiple model.

CR’s sex, CG’s and CR’s age, living situation, CG’s received support, relationship, educational attainment, duration of care, total care time, and benefits were not significantly associated with QoL. When entering CG’s age as a covariate into the initial multiple model, an additional significant effect of age on QoL was revealed; thus, CG’s age was included into the final model.

The results of a univariate covariance analysis (ANCOVA) with QoL as the independent variable, ‘Dementia’ as a fixed factor and CG’s sex, age, depressiveness score and employment status as covariates are shown in Table 3.

**Table 3:** ANCOVA Results for QoL by ‘Dementia’ Controlling for the Covariates Sex CG, Age CG, Depressiveness and Employment Status *(N = 386)*

	*SS*	*dF*	*F*	*p*	*partial η* ^*2*^
Dementia	*17.14*	*1*	*4.54*	*0.034*	*.012*
Depressiveness (*PHQ-9*)	*1292.03*	*1*	*342.21*	*< 0.001*	*.474*
Sex CG	*1.82*	*1*	*0.48*	*0.489*	*.001*
Age CG	*25.17*	*1*	*6.67*	*0.010*	*.017*
Employment status	*30.89*	*1*	*8.18*	*0.004*	*.021*
Error	*1434.72*	*380*	*3.78*		

*PHQ-9* Patient Health Questionnaire-9, *SS* Sum of Squares, *dF* Degrees of Freedom, *partial η*^*2*^ proportion of the total variability in *QoL* attributable to a factor or covariate (effect size measure); *R*^*2*^ *= .513 (Adj. R*^*2*^ *= .507)*

There was a significant effect *(p = 0.034)* of the factor ‘Dementia’ on QoL even after controlling for the effects of age, gender, depressiveness and employment status, suggesting that caregiving for a dementia patient was associated with lower levels of QoL in informal CGs.

## Discussion

In the present study, differences in health-related outcomes between dementia and non-dementia CGs were examined, based on a fairly large sample of CGs caring for someone legally considered to be dependent on care in Bavaria. By handing out the questionnaires directly upon the application for care level evaluation at the Medical Service of Compulsory Health Insurance Funds of Bavaria (MDK), every CG applying for an initial grade or upgrade of the care level for the CR at the Medical Service of Compulsory Health Insurance Funds of Bavaria (MDK) was addressed. In this way, systematic biases often associated with the acquisition of CGs were prevented for the recruitment of this subgroup of CGs.

Results demonstrate significant differences between the two groups of CGs regarding various health-related variables. CGs of elderly people with dementia reported a considerably high level of subjective burden, significantly higher than in the non-dementia group, which is consistent with other research on dementia caregiving and perceived strain (e.g. [20, 38]). Along the same lines as findings suggesting more psychological morbidities in dementia CGs (e.g. [10, 39]), in our study those caring for someone with dementia tended to suffer from higher levels of depressiveness, even though the difference was not statistically significant. Despite these findings, CGs in both groups experienced positive aspects of caregiving. Contrary to our expectations based on previous literature [40] physical complaints were more or less equally pronounced in both CG groups. Furthermore, bivariate comparisons showed that dementia CGs are more involved in caregiving in terms of the hours per day that they spend on caregiving tasks (e.g. ADL and IADL), which is similar to findings by Langa and colleagues [9].

To our knowledge this study is the first to examine differences in care-related QoL between CGs of dementia patients and those caring for patients with other chronic diseases using data from a fairly large sample of CGs. Not only did bivariate comparisons show significantly lower levels of care-related QoL in dementia CGs compared to non-dementia CGs, multivariate analysis also demonstrated that these lower QoL levels appear to be due to the different experiences that dementia CGs have with caregiving rather than to CG characteristics (e.g. sex, age, employment status) or other indicators of mental health (e.g. depressiveness). Even though the effect size of dementia status as a predictor is rather small, there seems to be something unique about caring for dementia patients, over and above from sociodemographics, objective characteristics of the care situation and mental health, which leads to a poorer QoL associated with caregiving.

In addition, the results of the multiple analysis showed a strong association between higher levels of CG depressiveness and lower care-related QoL, controlling for the impact of dementia status, which corroborates previous similar findings by Santos and colleagues [25] and Farina and colleagues [30]. Further factors related to higher levels of care-related QoL seem to be an older age and being unemployed. This suggests that work might play an important role in the development of stress and adverse health outcomes, either by constituting an additional stressor or as a result of a job-caregiving-conflict as explained by Pearlin (1990) in his CG stress model.

In line with previous literature, results of the current study showed that caregiving for an elderly person with dementia is associated with greater objective (e.g. total care time) and subjective strain, combined with greater depressiveness scores and a worse overall care-related QoL compared to non-dementia CGs. Although the present study provides valuable insight into the differences between dementia and non-dementia CGs, there are some limitations that must be considered when interpreting the results of this study.

### Limitations

One point of criticism of this study lays in the fact that due to high intercorrelations between subjective burden and depressiveness, only the depressiveness score was included as a covariate in the multiple prediction model for care-related QoL. Thus, the impact of dementia status on CGs’ care-related QoL has not been controlled for the level of perceived burden. To rule out this putative association, future research should include perceived burden as an additional impact factor when examining the influence of dementia status on care-related QoL in multiple models.

Another limitation is that it was not possible to assess all of the variables that may have an influence on CGs’ outcome variables, notably care-related QoL. With reference to Pearlin’s (1990) model of CG stress, in particular secondary stressors (e.g. job-caregiving conflict) and potential moderator variables (i.e. social support) have been largely ignored. In terms of objective strain, behavioral disturbances in particular are known to typically occur as dementia progresses and have been proven to be closely correlated with CG’s QoL levels [28]. Hence, these factors should be taken into consideration in future research so as to gain a more detailed and comprehensive understanding of the impact of dementia status on CGs’ QoL.

As the survey questionnaires were solely handed out to CGs addressing the MDK for the care level application, our study sample is limited to a subgroup of CGs and may not represent the whole population of informal CGs, Also, with a response rate of 26.1% the study sample did not reflect the wider sample, but only the selection of CGs who were motivated to participate in the survey and able to complete the questionnaire independently. Therefore the generalizability of our findings is limited. However, another procedure would not have been feasible without a violation of the ethical principle of informed consent. Future studies of this issue should be carried out with a more comprehensive sample of CGs.

Similar to the majority of other studies in this field, our data are cross-sectional rather than longitudinal, and thus, causal conclusions need to be drawn with caution. Results from longitudinal data would be necessary to establish a more comprehensive picture of the impact of caregiving on CGs’ care-related QoL when comparing dementia and non-dementia CGs. Finally, it must be noted that all data was obtained using self-report instruments, which constitutes a potential source of biases.

## Conclusions

The present study has some remarkable strengths. A large number of previous studies focusing on this issue have relied on rather small convenience samples that were recruited primarily from CG support groups or Alzheimer’s associations. Data for this study were obtained from a fairly large sample that is representative for CGs applying for an initial grade or upgrade of the care level for the CR at the Medical Service of Compulsory Health Insurance Funds of Bavaria (MDK) for someone legally considered to be dependent on care in Bavaria.

The current study corroborates findings from previous research, suggesting poorer health-related outcomes like greater subjective burden, higher levels of depressiveness and lower care-related QoL levels among dementia CGs.

In addition, this study is the first to establish that lower care-related QoL levels in informal CGs are due to experiences associated with caring for dementia patients rather than CG characteristics or mental health. Still, it remains largely unclear what exactly is associated with the status of being a dementia CG that leads to such adverse effects.

### Implications for policy and practice

Nevertheless, the key take-away of this study is that it is not appropriate to generalize findings from studies examining the impact of caregiving on informal CGs caring for elderly people without dementia to dementia CGs and vice versa. Data from this study indicate that caring for a dementia patient is not only associated with greater levels of CG burden and poorer mental health, but also with lower care-related QoL compared to non-dementia CGs. In spite of these findings suggesting a greater need for support for dementia CGs, in our sample CGs in the dementia group did not receive more formal or informal support than those in the non-dementia group, despite spending a greater amount of time on caregiving. It is therefore of immediate concern to encourage dementia CGs’ utilization of existing CG support services, but also to provide more specialized dementia care services, based on the specific stressors, challenges and needs associated with caregiving for elderly people with dementia. As our study revealed an association between greater depressiveness levels and decreased levels of QoL in CGs, professionals who come into contact with CGs (e.g. in CG counselling centres) should be on the lookout for CGs exhibiting depressive symptoms. In this respect, increased attention should be directed to dementia CGs once again, as they tend to show greater levels of depressiveness compared to non-dementia CGs, and therefore constitute the most vulnerable subgroup. As CG employment and a younger age seem to have adverse effects on QoL levels, a further focus should be placed on CGs facing the challenge of reconciling their job and caregiving activity, particularly when they are young.

For designing tailored intervention programs for dementia care, further research on potential causes of adverse effects of dementia caregiving as well as mediating and moderating impact factors on health itself would be valuable. Furthermore, longitudinal data would be needed to evaluate the effectiveness of potential interventions.

## Abbreviations

ADL: activities of daily living
BIZA: Berlin Inventory of Caregivers’ Burden - Dementia
CarerQoL: CarerQoL-7D
CG: caregiver
CR: care recipient
CSI: Caregiver Strain Index
GBB: Giessen Symptom List
IADL: instrumental activities of daily living
MDK: Medical Service of Compulsory Health Insurance Funds
PHQ-9: Patient Health Questionnaire-9
QoL: quality of life
SHI: statutory health care insurance
WHO: World Health Organisation

## Footnotes

1 The care level describes the extent to which care is needed on a 4-level ordinal scale: 0 (no care needed), 1 (mild care needed), 2 (moderate care needed), 3 (severe care needed). It is assessed by trained experts who are independent of the insurance system. Classification is based on the need for physical care. Formal care is financed by long-term care insurance on the basis of the care level.

2 The MDK is the official consulting and expertizing service for the statutory health and nursing care insurance (SHI). The SHI is the standard national health care insurance and covers over 85% of the German population.

3 Total daily care time consists of the daily time required for help with ADL, IADL and supervision of the CR

4 The median absolute deviation is a measure of statistical dispersion and is computed by averaging absolute differences between individual scores and the median of the variable. It is a more robust estimator compared to standard deviation, which is more resilient to outliers.

5 the general experience of happiness is a broad outcome measure that does not only pertain to caregiving, but may also include aspects of well-being that are unrelated to the caregiving activity, which makes it insensitive for the assessment of *care-related* QoL

6 two distinct scales with two scores which cannot be summed up into one comprehensive score for care-related QoL; due to the questionable benefit of a single-item VAS and to prevent potential dropouts related to CG’s confusion about differing answer formats within the questionnaire (VAS vs. ordinal scale) the CarerQoL-VAS was omitted
